# Significant functional impairment and disability in individuals with psoriatic arthritis and Achilles tendon pain: a cross-sectional observational study

**DOI:** 10.1007/s00296-024-05629-x

**Published:** 2024-06-08

**Authors:** Aimie Patience, Martijn Steultjens, Stefan Siebert, Gordon Hendry

**Affiliations:** 1 Musculoskeletal Health Research Group, School of Health and Life Sciences, Glasgow Caledonian University, Glasgow, UK; 2 MSK Podiatry, NHS Ayrshire and Arran, Kilmarnock, UK; 3 School of Infection and Immunity, University of Glasgow, Glasgow, UK

**Keywords:** Psoriatic arthritis, Achilles tendon, Enthesitis, Tendinopathy, Ultrasound

## Abstract

The Achilles tendon (AT) insertion is the most common site of enthesitis in psoriatic arthritis (PsA). The structure and function of the AT in PsA, and the prevalence of mid-portion pathology, is unknown. To compare the structure and function of the AT in people with PsA with self-reported AT pain (PsA + AT), PsA without self-reported AT pain (PsA-AT) and healthy controls. A cross-sectional, observational study was conducted. The ATs were assessed by clinical and US examination (B-mode and Power Doppler), performance-based testing (bilateral heel raise test (HRT) and 10 m walk test), and patient-reported outcome measures (PROMs) (including the Victorian Institute of Sport Assessment-Achilles [VISA-A]). Between-group differences were described using descriptive statistics, Chi-squared testing, parametric (1-way ANOVA) and non-parametric (Mann-Whitney or Kruskal-Wallis) testing. 22 PsA (11 per group) and 11 healthy control participants who were comparable in terms of sex, age, and BMI (PsA-AT = longer PsA disease duration) were recruited. VISA-A scores were significantly worse in the PsA + AT group compared to the PsA-AT group and healthy controls (*p < 0.001*). Inflammatory US features were significantly more prevalent in the PsA + AT group (*p < 0.001*). Mid-portion AT pathology was observed in the PsA + AT group, irrespective of entheseal disease. Clinical examination alone missed 5/7 cases of ‘active’ US-confirmed AT enthesitis. AT functional deficits were significant in the PsA + AT group and both PsA groups had lower HRT repetition rates and walked slower compared to healthy controls. Less than 1/3 of the PsA + AT group had received podiatry or physiotherapy care. Significant differences in the structure and function of the AT in PsA were noted. Despite management in line with current guidance, AT pain appears to persist and can result in severe functional impairment.

**Supplementary Information:**

The online version contains supplementary material available at 10.1007/s00296-024-05629-x.

## Introduction

Enthesitis, which presents as pain, swelling and inflammation at the insertion sites of tendons and ligaments to bone, is a hallmark clinical feature of psoriatic arthritis (PsA) with the insertion of the Achilles tendon (AT) to the posterior calcaneum the most common site affected [1]. The presence of enthesitis has been shown to be associated with increased levels of pain and disability, poorer quality of life and increased use of non-steroidal anti-inflammatory drugs (NSAIDs) and opioids [2]. Entheseal tissues may also play a role in the pro-inflammatory response to external stress and a possible link between repeated physical trauma and flare in PsA has previously been highlighted [3, 4]. Early identification and tight control of PsA disease activity, including enthesitis, is vital to prevent long-term functional impairment [5, 6].

The AT enthesis is often regarded as the archetypal ‘enthesis organ’ which includes the AT, retrocalcaneal bursa, Kager’s fat pad, calcaneal bone, fibrocartilage, and surrounding synovium [7]. Due to the location and weight-bearing nature of the AT, this tendon is prone to injury and micro-damage, even in healthy populations [8]. However, in contrast to enthesitis in PsA, in healthy populations the mid-portion of the AT (2–6 cm proximal to the insertion) is the most prevalent site of pathology [9, 10]. The prevalence and impact of mid-portion AT pathology in PsA has not yet been reported.

Clinical assessment of AT enthesitis in PsA has shown to correlate poorly with ultrasound (US) findings [11–13]. The Leeds Enthesitis Index (LEI) is the most widely used clinical enthesitis index validated in PsA and includes the AT but concerns around the sensitivity and specificity of the LEI have been reported [14, 15]. Clinical examination of AT enthesitis assesses pain on palpation at the AT insertion but cannot discriminate between inflammatory or structural pathology [12, 16]. It is also unclear whether clinical examination can differentiate between AT enthesitis and biomechanically-driven AT tendinopathy in a PsA population. Importantly, the management of enthesitis (NSAIDs, physiotherapy, corticosteroid injection, conventional disease-modifying anti-rheumatic drugs (csDMARDs) and biologics (bDMARDs)) [6, 17] differs greatly from the evidence-base and recommendations for more biomechanically-driven AT tendinopathy (eccentric exercises) [18].

US has shown good sensitivity and specificity for assessing features of AT enthesitis in PsA [11–13, 16, 19]. There are, however, a range of definitions and scoring criteria available for US assessment of AT enthesitis in PsA [20]. The latest OMERACT consensus-based definitions and scoring criteria are the first to group enthesitis features into either inflammatory (hypoechogenicity, increased tendon thickness and the presence of Doppler signal) or structural (enthesophytes/calcification and erosions) features [21]. To date, there are no evidence-informed treatment guidelines for AT enthesitis based on criteria derived from imaging-based outcomes i.e., the presence of structural or inflammatory changes. Individual US features of AT enthesitis also overlap with the US-based criteria for detecting insertional AT tendinopathy [22]. Due to the prevalence and pathogenesis of enthesitis in PsA, US research has largely focussed on entheseal pathology at the AT and the prevalence and impact of mid-portion AT tendinopathy has not yet been explored in PsA.

Despite a large number of studies highlighting the prevalence of AT enthesitis in PsA, very little has been published on the function of the AT in this population, which could be key to optimising care and lessening the burden of disease. Foot pain due to issues such as dactylitis, has been shown to reduce walking velocity in people with PsA [23, 24]. Woodburn et al. also reported reduced ankle power and AT force in a PsA cohort with US-confirmed AT enthesitis compared to healthy controls [25]. Exercise-based therapy has been advocated as a first-line treatment modality for enthesitis however PsA-related foot pain can be a barrier to physical activity but can also, significantly affect quality of life more generally [6, 26, 27].

This cross-sectional observational study aimed to compare AT structure and function, by utilising US imaging and performance-based testing, in people with PsA, with and without AT pain, compared to healthy controls. Greater understanding of both AT pathology and associated impairments in people with PsA with and without AT pain may help to inform more appropriate, targeted medical and non-medical management strategies in future.

## Materials and methods

### Sample population

This study compared 3 groups: people with PsA with AT pain, people with PsA without AT pain and age and sex-matched healthy controls. Adults aged ≥ 18 years with a clinician-diagnosis of PsA, with (PsA + AT) and without (PsA-AT) current uni/bilateral self-reported AT pain, were recruited from NHS Greater Glasgow and Clyde rheumatology outpatient clinics from January 2020 to February 2022. Participants were excluded if they reported a recent change in their systemic medication (within the last 6 weeks); intramuscular or local corticosteroid injection within the last 6 weeks; a history of AT rupture; a history of AT or major lower limb surgery; recent serious medical event or severe comorbid disease. Age and sex-matched healthy controls were recruited via Glasgow Caledonian University (GCU) staff email if they fulfilled the same criteria and had no history of an inflammatory arthropathy or self-reported AT pain.

Non-probability sampling was adopted, and participants were consecutively identified by the referring clinician (rheumatology health care professional). Written informed consent was obtained for all participants and the study was approved by the London Bridge Research Ethics Committee (19/LO/1139) and GCU School of Health and Life Sciences research ethics committee (HLS/PSWAHS/18/185) for PsA patients and healthy volunteers, respectively.

### Study protocol

Demographic data including age, sex, PsA disease duration, smoking status and current medications were collected using a standardised questionnaire. Patient-reported outcome measures (PROMs) included the Victorian Institute for Sports Assessment – Achilles (VISA-A) [28] questionnaire to assess the severity of AT symptoms ; the Health Assessment Questionnaire – Disability Index (HAQ-DI) [29] questionnaire to assess self-reported functional ability; the PsA Impact of Disease 12-item (PsAID-12) [30] questionnaire to assess PsA disease-related quality of life; the Hospital Anxiety and Depression Scale (HADS) [31] to assess levels of anxiety and depression; current and average AT pain (over the last 7 days) was reported using 0–10 pain visual analogue scale (VAS) and in the absence of a PsA-specific tool, the Rheumatoid Arthritis Foot Disease Activity Index-5 (RADAI-F5) [32] was used to measure foot disease activity.

### Clinical assessment of AT

Clinical assessment was performed by the lead researcher (AP - podiatrist) prior to US examination. The LEI was conducted to clinically assess for the presence of pain/tenderness on palpation at each AT enthesis. Subjective clinical assessments included the presence of current self-reported pain in the ATs and self-reported morning stiffness at the AT. Pain during passive dorsiflexion of the AT (with the knee flexed) and resisted plantarflexion of the ankle was also assessed.

### US assessment of AT

The European Alliance of Associations for Rheumatology (EULAR) recommendations for the reporting of US studies in rheumatic and musculoskeletal diseases (RMDs) were used to guide the reporting of US procedures and results [33]. A CASE-trained (Consortium for the Accreditation of Sonographic Education postgraduate certificate) US practitioner (AP) with 3 years of scanning experience performed all US examinations. The 2017 EULAR standardised procedures for US imaging in rheumatology were followed [34]. Participants were positioned prone with both feet in passive plantarflexion. The ATs were scanned in both longitudinal and transverse views and both static images and dynamic cine-loops were recorded. Image acquisition and US reporting was performed by the same US practitioner during a single study visit.

All scanning was performed on the same GE LOGIQ S8 US machine in the same consulting room using a multi-frequency linear transducer (L6-15 MHz). Conventional greyscale (B-mode) and Power Doppler imaging were utilised to assess the full length of the ATs. Power Doppler settings were adjusted (pulse repetition frequency, gain, box size/positioning) for each participant and the pressure on the tendons from the transducer was minimised to increase sensitivity and reduce the presence of artefacts [35].

The OMERACT consensus-based definitions of features of enthesitis on US were adopted [21]. Power Doppler signal at the entheses was scored from 0 to 3 (0 = No Doppler signal, 1 = < 2 punctiform (discrete areas) Doppler signal with no confluent (merging together) Doppler signal, 2 = 2–4 punctiform Doppler signal or 1 confluent Doppler signal and 3 = > 4 punctiform Doppler signals or > 1 confluent Doppler signal) based on OMERACT recommendations [36]. The anteroposterior diameter of the AT was measured in both longitudinal and transverse planes at 3 locations: (1) the insertion of the deep margin of the AT to the calcaneal bone, (2) the thickest part of the mid-portion (2–6 cm from insertion), and (3) the soleus myotendinous junction [37].

### AT function

Functional endurance of the AT was measured using the bilateral heel raise test [38]. Participants were instructed to lift their heels and rise onto their forefoot with both feet on the ground, with the option of holding on to the wall for stability. When participants reached maximum plantarflexion, the researcher marked the participants’ height (top of head) using an adjustable height chart bar. Participants performed as many heel-raises as they could (with the top of their head reaching the bar) until the point of self-determined fatigue. If participants were unable to do any heel raises the score was recorded as zero. No encouragement was given throughout the test. Speed of repetitions was self-selected, and the repetition rate (repetitions per second) was recorded.

Gait speed was assessed by the 10-metre walk test (10MWT). A 14-metre walkway was set up with 2 m to warm up and 2 m at the end to slow down to a stop [24]. Participants were instructed to walk at a ‘comfortable pace’ and could rest between the two walking trials. If required, participants were permitted to use a walking aid device for stability. The average speed (in seconds) of the two walking trials was recorded.

### Statistical analysis

Statistical analyses were performed using SPSS version 26 (IBM Corp, Armonk, NY, USA). Descriptive statistics were used to describe demographic, clinical, US, and performance-based data as mean (standard deviation (SD)), median (inter-quartile range (IQR)), or absolute (n) and relative frequencies (%). Between group differences were described using Chi-squared testing (*X*^2^), parametric (1-way ANOVA and post-hoc testing) and non-parametric (Mann-Whitney (*U*) or Kruskal-Wallis (*H*)) testing where appropriate (statistical significance was defined as *p* ≤ 0.05). Using one-way ANOVA to evaluate differences between groups, based on an anticipated effect size of 0.55, *p* = 0.05 significance and 0.8 power, we aimed for 12 participants in each group (total *n* = 36). As no follow-up was involved, it was not necessary to account for attrition.

## Results

A total of 33 participants were recruited to the study, with 11 in each (Table 1)group. There were no significant differences in sex (*H*(2) = 0.241, *p* = 0.887), age (*H*(2) = 2.119, *p* = 0.347) or body mass index (BMI) (*H*(2) = 3.619, *p* = 0.164) between the three groups. The PsA-AT group had a significantly higher disease duration than the PsA + AT group (*U* = 95.5, *p* = 0.019) but age (*U* = 80.5, *p* = 0.193) and BMI scores (*U* = 30.0, *p* = 0.152) were not significantly different between these two PsA groups. In the PsA + AT group, the duration of AT symptoms varied (median 2 years, range 0.25-20 years) and 3/11 participants experienced AT symptoms prior to their PsA diagnosis (range 2–6 years).

**Table 1:** Participant characteristics

	PsA + AT (*n* = 11)	PsA-AT (*n* = 11)	Healthy controls (*n* = 11)	*p* value
**Participant characteristics**				
Sex (female)	7/11 (64%)	6/11 (55%)	6/11 (55%)	0.887
Age (years)	49 (23)	54 (16)	52 (14)	0.347
BMI (kg/m^2^)	29.6 (12.3)	25.4* (5.1)	24.9 (8.3)	0.164
Disease duration (years)	7.4 (6.3)	20.1 (13.1)	-	**0.019**
Currently smoking	1/11 (9.1%)	0/11 (0%)	0/11 (0%)	-
Previous smoking	4/11 (36.4%)	6/11 (54.5%)	2/11 (18.2%)	-
**Current medication**				
NSAIDs	0/11 (0%)	1/11 (9.1%)	-	-
csDMARDs	3/11 (27.3%)	4/11 (36.4%)	-	-
bDMARDs	6/11 (54.5%)	6/11 (54.5%)	-	-
Combination bDMARD/csDMARD	1/11 (9.1%)	0/11 (0%)	-	-

PsA + AT = PsA with AT pain group, PsA-AT = PsA without AT pain group, healthy controls. Data presented as mean (standard deviation). BMI = body mass index. NSAIDs = non-steroidal anti-inflammatory drugs. csDMARDs = conventional synthetic disease-modifying anti-rheumatic drugs. bDMARDs = biologic drugs. Kruskal-Wallis test for comparison of all groups, Mann-Whitney U test for disease duration comparison between PsA groups.**Bold** indicates a statistically significant difference (*p* < 0.05). *BMI data only available for *n* = 9/11 in PsA-AT group.

In line with the study design, there was a significant association between group allocation and the presence of AT involvement on LEI (*X*^2^(2) = 14.67, *p* = 0.001), self-reported current AT pain (*X*^2^(2) = 33.0, *p* < 0.001), self-reported AT morning stiffness (*X*^2^(2) = 21.21, *p* < 0.001), pain on passive dorsiflexion of the ankle (*X*^2^(2) = 29.07, *p* = 0.011) and pain on resisted plantarflexion of the ankle (*X*^2^(2) = 2.06, *p* = 0.003) (Table 2). Self-reported AT pain VAS scores were significantly different between groups for both current AT pain (*H*(2) = 19.78, *p* < 0.001) and average AT pain over the last 7 days (*H*(2) = 20.5, *p* < 0.001). There were no significant differences between groups for the 10MWT but pairwise comparison testing showed the heel raise repetition rate was significantly different between PsA groups (*p* = 0.005) and between PsA participants and healthy controls (*U* = 53.0, *p* = 0.008).

**Table 2:** Comparison of clinical and functional AT assessments

	PsA + AT (*n* = 11)	PsA-AT (*n* = 11)	Healthy controls (*n* = 11)	*p* value
**Self-reported**				
Presence of AT morning stiffness	10 (90.9%)	1 (9.1%)	1 (9.1%)	**< 0.001**
VISA-A (0-100)	35.8 [25.7]	70.8 [18.9]	92.3 [7.3]	**< 0.000**
Current AT pain VAS (0–10)	4.8 [5.7]	0 [0]	0 [0]	**< 0.001**
AT pain VAS in last 7 days (0–10)	3.6 [6.3]	0 [0]	0 [0]	**< 0.001**
**Clinical assessment**				
Clinical AT enthesitis (LEI)	6 (45.5%)	0	0	**0.001**
Pain on palpation	11 (100%)	1 (9.1%)	0	**< 0.001**
**Tendon loading**				
Pain on passive dorsiflexion	4 (36.4%)	0	0	**0.011**
Pain on resisted plantarflexion	5 (45.5%)	0	0	**0.003**
**Performance-based measures**				
Heel raise rep rate	0.72 [0.67]	1.24 [0.83]	1.29 [0.42]	**0.005**
10 MWT time (seconds)	13.87 [5.63]	12.48 [4.98]	11.62 [2.12]	0.086

PsA + AT = PsA with AT pain group, PsA-AT = PsA without AT pain group. Data presented as median [interquartile range]. p values based on Pearson’s chi squared statistical test, **bold** indicates *p* < 0.05 for significance.

### Ultrasound features

The presence of ‘active’ enthesitis (2018 OMERACT definition [21]), was detected in 7/22 AT entheses (5 participants, including 2 with bilateral) in the PsA + AT group (Table 3). Data regarding US features in participants is also presented (Supplementary Table 1.) No participants in the other two groups had US evidence of active enthesitis. ‘Non-active’ enthesitis, i.e. US enthesitis features but no Power Doppler signal, was detected across all three groups, with no significant differences between groups.

**Table 3:** US characteristics

	PsA + AT (*n* = 22 ATs)	PsA-AT (*n* = 22 ATs)	Healthy controls (*n* = 22 ATs)	*p* value	*p* value (PsA + AT + v -AT)
Active enthesitis	7 (31.8%)	0	0	**< 0.001**	**0.004**
Non-active enthesitis	6 (27.3%)	7 (31.8%)	3 (13.6%)	0.342	0.714
**Inflammatory features**					
Hypoechogenicity	13 (59.1%)	3 (13.6%)	3 (13.6%)	**0.001**	**0.002**
Increased thickness	9 (40.9%)	5 (22.7%)	1 (4.5%)	**0.016**	0.195
Power Doppler	7 (31.8%)	0	0	**< 0.001**	**0.004**
**Structural features**					
Enthesophytes	15 (68.2%)	11 (50%)	4 (18.2%)	**0.003**	0.220
Calcification	10 (45.5%)	9 (40.9%)	2 (9.1%)	**0.019**	0.761
Erosion	8 (36.4%)	7 (31.8%)	2 (9.1%)	0.086	0.750
Retrocalcaneal bursitis	2 (9.1%)	4 (18.2%)	0	0.111	0.380
**Power Doppler enthesis**					
Grade 0	15	22	22	**< 0.001**	**0.004**
Grade 1	7	0	0	**< 0.001**	**0.004**
Grade 2 or 3	0	0	0	-	-
**AT thickness (mm)**					
Enthesis (Range)	5.6 [1.7] 3.6–9.6	4.6 [0.8] 3.0-6.9	4.4 [0.7] 3.1-6.0	**0.003**	-
Mid-portion (Range)	6.1 [2.5] 4.1–16.1	5.3 [1.0] 3.8–7.5	5.5 [1.2] 4.8–8.5	0.239	-
Myotendinous (Range)	4.5 [0.9] 3.2-7.0	4.1 [0.7] 3.1–6.2	4.1 [0.7] 3.4–5.9	0.142	-

Data presented as n (%) of entheses. AT thickness presented in mm as mean [SD]. AT = Achilles tendon; PsA + AT = PsA with AT pain group (*n* = 22 tendons), PsA-AT = PsA without AT pain group (*n* = 22 tendons), healthy controls (*n* = 22 tendons). p values based on Pearson’s chi squared statistical test, bold indicates *p* < 0.05 for significance.

While individual inflammatory features of hypoechogenicity and increased thickness were also noted in some PsA and healthy participants without AT pain, these were much more common in those with PsA + AT while Power Doppler was only noted in the PsA + AT group. Individual structural US features were noted in both PsA groups, regardless of presence of current AT pain, and significantly more often than in healthy controls. The most common structural US finding in healthy controls was the presence of enthesophytes. The main and only significant difference in AT thickness on US was increased AT thickness at the entheseal insertion in those with PsA + AT.

Inflammatory and structural US changes were more common in the PsA + AT group. Relative to the healthy control group, both PsA groups exhibited more structural features of entheseal pathology. Inflammation identified by Power Doppler imaging was only detected in the PsA + AT group.

**Table 4:** Comparison of US characteristics in PsA participants with clinical AT enthesitis detected on LEI

US feature	Clinical AT enthesitis (*n* = 9)	No clinical AT enthesitis (*n* = 35)	*p* value
Active enthesitis	2 (22.2%)	5 (14.3%)	0.562
Non-active enthesitis features	6 (66.7%)	7 (20.0%)	**0.006**
Inflammatory features			
Hypoechogenicity	8 (88.9%)	8 (22.9%)	**< 0.001**
Thickening	7 (77.8%)	7 (20.0%)	**0.001**
Power Doppler	2 (22.2%)	5 (14.3%)	0.562
Structural features			
Calcification	6 (66.7%)	13 (37.1%)	0.111
Enthesophytes	9 (100%)	17 (48.6%)	**0.005**
Erosions	7 (77.8%)	8 (22.9%)	**0.002**
Retrocalcaneal bursitis	1 (11.1%)	5 (14.3%)	0.805
AT thickness (mm)			
Entheses	6.48 [1.3]	4.75 [1.2]	**< 0.001**
Mid-portion	5.42 [0.8]	5.78 [2.1]	0.909
Myotendinous	4.52 [0.4]	4.29 [0.9]	0.104

Clinical enthesitis scored as present based on the Leeds Enthesitis Index (LEI) AT sub-scale. p values based on Pearson’s chi squared statistical test, **bold** indicates *p* < 0.05 for significance. AT thickness presented as mean [SD]. p values for AT thickness from Mann-Whitney U test for significance.

Of the 22 participants with PsA, AT enthesitis was detected on LEI in 9 entheses (6 participants with PsA + AT). The association of US findings with LEI is presented in Table 4. The presence of non-active enthesitis features (*X*^2^(1) = 7.49, *p* = 0.006), inflammatory features (*X*^2^(1) = 9.634, *p* = 0.002) including hypoechogenicity ( *X*^2^(1) = 13.489, *p* < 0.001) and entheseal thickening *X*^2^(1) = 11.016, *p* = 0.001), structural features (*X*^2^(1) = 5.852, *p* = 0.016) including enthesophytes (*X*^2^(1) = 7.833, *p* = 0.005) and erosions (*X*^2^(1) = 9.610, *p* = 0.002) were significantly different between groups. For AT thickness, only entheseal thickness measurements were significantly different between groups (*U* = 275, *p* < 0.001).

**Table 5:** Comparison of PROM scores across all groups

PROM	Group	*n*	Mean	SD	Range	*p* value
PsAID (0–10)	PsA + AT	10	5.52	1.76	2.3–7.8	0.018*
	PsA -AT	10	3.38	1.92	0.5–5.9	
	Healthy controls	0	-	-	-	
HAQ-DI (0–3)	PsA + AT	11	1.21	0.76	0.00–2.38	**0.001**
	PsA -AT	11	0.76	0.84	0.00–2.38	
	Healthy controls	11	0.57	0.11	0.00–2.38	
HADS-A (0–21)	PsA + AT	11	6.82	5.02	1–14	0.337
	PsA -AT	11	7.82	3.63	4–15	
	Healthy controls	11	5.09	4.16	0–12	
HADS-D (0–21)	PsA + AT	11	6.64	4.00	1–13	**0.001**
	PsA -AT	11	6.09	3.56	0–11	
	Healthy controls	11	1.27	2.33	0–8	
RADAI-F5 (0–10)	PsA + AT	10	5.20	3.27	1.0–8.6	**< 0.000**
	PsA -AT	6	1.20	1.20	0.2–3.2	
	Healthy controls	10	0.80	0.17	0.0–0.4	

PROM = patient reported outcome measure. PsA + AT = PsA with AT pain group, PsA -AT = PsA without AT pain group. *n* < 11 indicate missing data. PsAID = Psoriatic Arthritis Impact of Disease questionnaire, HAQ-DI = Health Assessment Questionnaire – Disability Index, HADS-A/D = Hospital Anxiety and Depression Scale – Anxiety & Depression scores, RADAI-F5 = Rheumatoid Arthritis Foot Disease Activity Index-5. n = number, SD = standard deviation, p-value from one-way ANOVA, **bold** = *p* = 0.05 for significance.

PROMs in the three groups were assessed although PsAID-12 (impact of PsA on quality of life) was only assessed in PsA participants (Table 5). The presence of self-reported AT pain was significantly associated with higher PsAID-12, HAQ-DI and RADAI-F5 scores indicating a negative impact on quality of life and foot disease activity. In contrast, the anxiety and depression scores were higher in participants with PsA compared to healthy controls, regardless of the presence or absence of self-reported AT pain.

Table 6. presents a summary of the clinical and demographic data for the PsA + AT group. Only 3/11 participants had received, or were waiting to receive, treatment for their AT pain/pathology.

**Table 6:** PsA with AT pain group description

Participant	AT/PsA information				US features		Performance-based measures	
	AT duration (years)	PsA duration (years)	Medication	AT treatment history	US inflam (0–6)	US struc (0–6)	HRT (rep rate)	10 MWT (seconds)
1	10	4	Combo	Physio + pod	0	0	0.97	7.66
2	0.25	4	csDMARD	None	0	0	0.72	12.67
3	2	5	bDMARD	None	5	1	0	29.88
4	1	8	bDMARD	None	2	4	0.56	13.80
5	1	2	bDMARD	None	4	5	0.72	12.50
6	20	20	None	None	4	5	0.84	14.16
7	4	1	csDMARD	Awaiting	6	6	0.97	13.87
8	7	5	bDMARD	Pod	5	5	0.29	18.14
9	1	6	bDMARD	None	0	0	0.49	11.33
10	2	7	bDMARD	None	0	3	1.17	15.57
11	2	19	csDMARD	None	3	4	0	47.29

Data presented from PsA group with AT pain (*n* = 11). AT duration = duration of AT pain. Medication: csDMARDs = conventional disease-modifying anti-rheumatic drugs, bDMARDs = biologic disease-modifying anti-rheumatic drugs. Physio = physiotherapist, pod = podiatrist. US inflam = number of inflammatory enthesitis features from the 2018 OMERACT definition (hypoechogenicity, increased thickness of enthesis and presence of Doppler), US struc = number of structural enthesitis features (enthesophytes, calcification and erosion). HRT = heel raise test, rep rate = repetition rate (reps/second). 10 MWT = 10 m walk test.

## Discussion

This study presents and brings together novel findings around the pathology, structure, and function of the AT in people with PsA with and without self-reported AT pain. The PsA + AT group showed significant AT functional impairment and lower PsA-related quality of life scores (assessed by the PsAID-12) compared to the PsA-AT group and healthy controls. While some US AT findings were noted in all groups, these were mainly structural findings in those without AT pain, with inflammatory features of enthesitis on US (hypoechogenicity, tendon thickening and Power Doppler signal) more prevalent in the PsA + AT group, although not in all.

The PsA + AT pain group had the highest number of features of AT pathology on clinical examination, and positive results for AT tendinopathy based on tendon loading tests (pain on passive dorsiflexion and pain on resisted plantarflexion) were only found in the PsA + AT group. However, this study adds to a body of evidence that suggests there is a poor correlation between clinical assessment for AT enthesitis (palpatory tenderness) and US findings [11–13, 16]. Importantly, clinical examination only picked up on 2/7 (28.6%) cases of US ‘active’ enthesitis (US enthesitis features with the presence of Doppler signal). Over 50% of AT entheses in the PsA-AT group had one or more structural feature of enthesitis (calcification, enthesophytes or erosion) on US examination and 30% had one or more inflammatory features (hypoechogenicity, thickening, Power Doppler signal). This potential lack of correlation between clinically-defined and US-defined AT enthesitis has been previously reported in a spondyloarthritis cohort where the authors suggested that differential diagnosis for clinically-defined AT enthesitis included subtalar joint arthritis, tibialis posterior tenosynovitis and retrocalcaneal bursitis [39]. In this study, mid-portion AT tendinopathic changes, intra-substance mid-portion tears and retrocalcaneal bursitis were observed on US in both PsA groups. This again highlights the potential clinical utility of using US to define the underlying pathology at the AT and use this to tailor treatment accordingly.

The OMERACT consensus-based, validated definitions and scoring of enthesitis features used in this study have been reported to have excellent inter and intra-reader agreement [21]. Individual enthesitis features were scored as present/absent and although the original OMERACT enthesitis scoring system does not include a score for Doppler signal, the presence of Power Doppler signal was scored from 0 to 3 in this study based on the updated 2019 OMERACT definitions for ultrasonographic pathologies and elementary lesions of RMDs [36]. Power Doppler signal was detected at 7 AT entheses (31.8%) in the PsA + AT group (15.9% PsA participants) and all scored as Grade 1 (< 2 punctiform Doppler signals with no confluent Doppler signal). Previous studies have reported similar incidences of Doppler activity at the AT enthesis in PsA (range = 6-36%) [40–44].

PsA disease duration was significantly higher (*p* = 0.019) in the PsA-AT group compared to the PsA + AT group and this could potentially explain the similar prevalence of structural enthesitis features on US. Alternatively, individuals with PsA could just exhibit more US enthesitis features that may be asymptomatic. This could suggest participants have experienced symptoms of AT enthesitis (or subclinical manifestations) earlier on in their disease course that has resulted in persisting structural change i.e., this may reflect the natural course of AT enthesitis in PsA. Despite a relatively short PsA disease duration (9/11 participants < 8 years), and 10/11 participants on either csDMARD or bDMARD therapy, the majority of the PsA + AT group had structural features of AT enthesitis on US. Although enthesophytes have been detected in ‘normal’ AT entheses in healthy populations and appear to be more prevalent with increasing age [44], no significant differences in age were noted between PsA groups. Healthy control participants in this study also exhibited both structural and inflammatory features of enthesitis at the AT at lower rates that those with PsA + AT and none exhibited Power Doppler signal. Although these structural US features were significantly more prevalent in the PsA cohort compared to controls, it does highlight the likely role of biomechanics and resultant micro-trauma on the ‘normal’ enthesis. As the presence of enthesophytes is common in healthy populations and appears to be associated with age, the clinical relevance of the presence of structural entheseal features on US in PsA has been questioned [39, 44].

Previous studies have reported changes in walking speed and gait parameters in PsA [24–26]. This study showed a reduction in average walking speed in both PsA groups compared to healthy controls, but the difference was not statistically significant (*p* = 0.086). This difference may also be due to involvement of other lower limb or axial joints in PsA, which were not formally assessed. In this study, AT function assessed using the heel raise test was significantly impaired in the PsA groups compared to healthy controls (*p* = 0.008). The PsA + AT group also scored significantly worse on the PsAID-12, HAQ-DI and RADAI-F5 compared to the PsA-AT group. However, in the absence of PsA-validated PROMs, the measurement properties of the RADAI-F5 for PsA populations is still unknown and this can also be affected by involvement of other joints in the foot and/or ankle beyond the AT.

In the PsA + AT group, three participants experienced AT symptoms prior to their PsA diagnosis, of which two exhibited the highest number of AT enthesitis features on US compared to the rest of the symptomatic group. These three participants with AT pain prior to their PsA diagnosis had either visited a physiotherapist or podiatrist for AT treatment, or were awaiting treatment, while the rest of the symptomatic cohort reported no physiotherapy or podiatry input. Inadequate access to foot care provision in PsA has previously been highlighted with only 20% of PsA patients with moderate-to-high levels of foot impairment and disability receiving foot care in the UK in 2010 [45] and similar barriers to accessing care reported in Australia and New Zealand in 2019 [27]. This study has also demonstrated that AT pathology can often extend beyond the enthesis in PsA and therefore identification of the origin of pain using US as a supporting tool, i.e., structural, inflammatory, or mid-portion pathology, could help guide more targeted therapy.

### Limitations

A limitation of the study is the small sample size and possibility of selection bias as we did not adopt random sampling. As a result, the US and clinical presentation of AT pathology in the PsA groups may not be representative of the PsA population. However, the prevalence of AT pathology in both PsA participants and healthy controls is in line with previously published US studies [11, 13, 43]. Based on the EULAR recommendations for the reporting of US studies in RMDs, we acknowledge some further US protocol-related limitations to our study [34]. Although there are advantages to the same US practitioner using the same US machine to perform all examinations, this meant the US practitioner was not blinded to the results of the clinical examination or group allocation. Although US image acquisition was standardised, there remains an element of subjectivity and US practitioner-interpretation with all US-based findings. We also did not control for contextual factors such as the ambient temperature [47], limiting physical activity prior to US examination or controlling the use of NSAIDs prior to examination, which could all influence Doppler imaging [48]. In the PsA + AT cohort, 3/11 reported their AT symptoms at the time of assessment were unilateral however US scores (B-mode and Power Doppler) were reported for both ATs. The decision to include both ATs in the symptomatic group was made based on previous research that has shown pathology can often occur at the contralateral limb but be asymptomatic [49, 50]. The study also adopted PROMs not yet validated in a PsA population, including the VISA-A.

## Conclusion

In summary, there were significant differences in the structure and function of the AT between groups which could be clinically relevant when assessing and managing AT pain in people with PsA, although the presence of these features in the general population also needs to be considered. The results of this study add to a body of literature suggesting AT pathology in PsA cannot be fully assessed by clinical examination alone. Although the prevalence of AT enthesitis in PsA is well-documented, our findings reveal that AT enthesitis and mid-portion Achilles tendinopathy may co-exist in people with PsA, or be misdiagnosed, which could affect the efficacy of treatment interventions. As treatment guidelines for AT enthesitis and AT tendinopathy vary greatly, and distinguishing these is complex, it is likely that PsA patients with symptomatic ATs may be receiving inappropriate, or suboptimal, care.

Assessments of AT function showed significant impairment in people with PsA compared to healthy controls, regardless of self-reported AT pain. Less than a third of participants had received treatment from podiatrist or physiotherapist for their AT symptoms, further highlighting the unmet need for foot and ankle care in PsA. Of note, PsA participants with AT pain in receipt of medical management in line with the current clinical guidelines appear to still be severely impacted by their AT pain despite these therapies. Robust research is required to address the evidence gap with regards to standardised assessment and non-pharmacological management of AT pathology in PsA.

### Electronic supplementary material

Below is the link to the electronic supplementary material.

Supplementary Material 1

## Abbreviations

10MWT − 10: Metre walk test
AT: Achilles tendon
bDMARDs: Biologic disease-modifying anti-rheumatic drugs
BMI: Body mass index
CASE: Consortium for the Accreditation of Sonographic Education
csDMARDs: Conventional disease-modifying anti-rheumatic drugs
EULAR: European Alliance of Associations for Rheumatology
GCU: Glasgow Caledonian University
HADS: Hospital Anxiety and Depression Scale
HADS: A-Hospital Anxiety and Depression Scale-Anxiety component
HADS: D-Hospital Anxiety and Depression Scale-Depression component
HAQ: DI-Health Assessment Questionnaire-Disability Index
HRT: Heel raise test
IQR: Inter-quartile range
LEI: Leeds Enthesitis Index
NSAIDs: Non-steroidal anti-inflammatory drugs
OMERACT: Outcome Measures in Rheumatology
PROMs: Patient-reported outcome measures
PsA: Psoriatic arthritis
PsA + AT: PsA with self-reported Achilles tendon pain
PsA: AT-PsA with no self-reported Achilles tendon pain
PsAID: 12-PsA Impact of Disease-12 items
RADAI: F5-Rheumatoid Arthritis Foot Disease Activity Index-5
RMDs: Rheumatic and musculoskeletal diseases
SD: Standard deviation
US: Ultrasound
VAS: Visual analogue scale
VISA: A-Victorian Institute for Sports Assessment-Achilles questionnaire
