# Supplementary material for: Significant functional impairment and disability in individuals with psoriatic arthritis and Achilles tendon pain: a cross-sectional observational study
Source: Rheumatol Int. 2024 Jun 8;44(8):1469–79. doi: 10.1007/s00296-024-05629-x (PMC11222213; doi:10.1007/s00296-024-05629-x)
Supplement: Supplementary file 1 — Supplementary Material 1 [file 296_2024_5629_MOESM1_ESM.docx]

|  | **PsA+AT**  (n=11) | **PsA-AT**  (n=11) | **Healthy controls**  (n=11) |
| --- | --- | --- | --- |
| Active enthesitis | (31.8%) | 0 | 0 |
| Non-active enthesitis | 6 (27.3%) | 7 (31.8%) | 3 (13.6%) |
| **Inflammatory features** |  |  |  |
| Hypoechogenicity | 7 (63.6%) | 2 (18.1%) | 2 (18.1%) |
| Increased thickness | 5 (45.5%) | 3 (27.3%) | 1 (9.1%) |
| Power Doppler | 5 (45.5%) | 0 | 0 |
| **Structural features** |  |  |  |
| Enthesophytes | 8 (72.7%) | 7 (63.6%) | 2 (18.1%) |
| Calcification | 7 (63.6%) | 6 (54.5%) | 1 (9.1%) |
| Erosion | 4 (36.4%) | 6 (54.5%) | 2 (18.1%) |
| Retrocalcaneal bursitis | 1 (9.1%) | 4 (36.4%) | 0 |
| *Data presented as n (%)* *of participants. AT thickness presented in mm as mean [SD]. AT = Achilles tendon; PsA +AT = PsA with AT pain group (n=11 participants), PsA-AT = PsA without AT pain group (n=11 participants), healthy controls (n=11 participants)* | | | |

**Supplementary Table 1. US characteristics per participant**
